# Screening for Anti-Influenza Actives of Prefractionated Traditional Chinese Medicines

**DOI:** 10.1155/2020/4979850

**Published:** 2020-10-14

**Authors:** Qianli Kang, Yanyan Wang, Qinghua Cui, Lili Gong, Yong Yang, Haiqiang Jiang, Lijun Rong, Rong Rong, Ruikun Du

**Affiliations:** ^1^College of Pharmacy, Shandong University of Traditional Chinese Medicine, Jinan 250355, Shandong, China; ^2^Shandong Provincial Collaborative Innovation Center for Antiviral Traditional Chinese Medicine, Jinan 250355, Shandong, China; ^3^Qingdao Academy of Chinese Medicinal Sciences, Shandong University of Traditional Chinese Medicine, Qingdao 266122, Shandong, China; ^4^Experimental Center, Shandong University of Traditional Chinese Medicine, Jinan 250355, Shandong, China; ^5^Department of Microbiology and Immunology, College of Medicine, University of Illinois at Chicago, Chicago 60612, IL, USA

## Abstract

Traditional Chinese medicines (TCMs) have proven to possess advantages in counteracting virus infections according to clinical practices. It's therefore of great value to discover novel antivirals from TCMs. In this paper, One hundred medicinal plants which have been included in TCM prescriptions for antiviral treatment were selected and prefractionated into 5 fractions each by sequentially using cyclohexane, dichloromethane, ethyl acetate, *n*-butanol, and water. 500 TCM-simplified extracts were then subjected to a phenotypic screening using a recombinant IAV expressing Gaussia luciferase. Ten TCM fractions were identified to possess antiviral activities against influenza virus. The IC50's of the hit fractions range from 1.08 to 6.45 *μ*g/mL, while the SIs, from 7.52 to 98.40. Furthermore, all the ten hit fractions inhibited the propagation of progeny influenza virus significantly at 20 *μ*g/mL. The hit TCM fractions deserve further isolation for responsible constituents leading towards anti-influenza drugs. Moreover, a library consisting of 500 simplified TCM extracts was established, facilitating antiviral screening in quick response to emerging and re-emerging viruses such as Ebola virus and current SARS-CoV-2 pandemic.

## 1. Introduction

Influenza A virus (IAV) infections usually cause acute respiratory disease with substantial morbidity and mortality [1]. According to the World Health Organization (WHO), seasonal influenza causes up to 650,000 deaths annually [2]. Vaccines and antivirals are effective countermeasures in combating IAV infections. However, current influenza vaccines require reformulation annually and do not always match circulating strains, while resistance to marketed anti-influenza drugs are increasingly emerging [3–5]. Therefore, novel antivirals with different mechanism of action are urgently needed.

Historically, natural products were the source of virtually all medicinal preparations, such as TCMs. More recently, natural products or their derivatives have continued to enter clinical trials, accounting for 32% of new medicines approved by the US Food and Drug Administration (FDA) between 1981 and 2014 [6]. However, traditionally natural-product research usually subject concentrated extract samples to screening. Such extracts are complicated mixtures, and all the components may reach the biological target in the assay, bringing several technical problems, including (i) the concentrations of some components are too low to have measurable effects; (ii) interference or nuisance compounds may confound the signal from the assay; and (iii) the possibility of additive or synergistic effects of several compounds [7]. It is unlikely to isolate each compound from a crude extract in advance, since it is too onerous and uneconomical. Alternatively, prefractionation strategies have been reported to solve, at least partially, these problems [8, 9]. For example, in a high-throughput extract prefractionation screening, 80% of the primary-screen hits from prefractionated samples were not observed in assays of the associated crude extract [10].

In the present study, 100 medicinal plants which have been included in TCM prescriptions for antiviral treatment were selected and prefractionated into 5 fractions each. The library consisting of 500 prefractionated TCM extracts was subsequently subjected to a phenotypic screening for anti-influenza actives [11, 12]. As a result, ten TCM fractions were identified to have antiviral potency against IAV, deserving further analysis for novel anti-influenza lead drugs.

## 2. Methods

### 2.1. Plant Materials

One hundred medicinal plants that have been previously used for antiviral treatment were purchased from Affiliated Hospital of Shandong University of Traditional Chinese Medicine (Jinan, China). The botanical authentication was performed by Pro. Lingchuan Xu (College of Pharmacy, Shandong University of Traditional Chinese Medicine, Jinan, China). Voucher specimens of these materials were deposited for references in our lab. The samples were stored at −20°C and pulverized before use. More information of the 100 plants are provided in Table S1

### 2.2. Standard Extraction Preparation

Each dry powder (200 g) of TCMs was extracted with 1000 mL cyclohexane (CYH) firstly, and then the leftover solution was sequentially extracted using dichloromethane (DCM), ethyl acetate (EAC), *n*-butanol (NBA), and distilled water (W), according to the method described by Liu et al [13, 14]. The five extracted solutions of 100 TCMs were harvested separately by ultrasonic extraction at 40°C for 30 min and repeated twice followed by filtration and evaporation under vacuum. The organic fractions were dried in 45°C vacuum, and the water fractions were freeze-dried. All 500 samples were sealed in vessels and refrigerated separately.

A portion of each extracts was removed, dissolved in dimethyl sulfoxide (DMSO) at 50 mg/mL, and arrayed in 96-well plates as a prefractionated TCMs library.

### 2.3. HPLC Separation Validation

To illustrate the stability of extraction method, several active prefractionations were selected for analysis by HPLC using a SunFire-C18 TM column (4.6 × 150 mm, 3.5 *µ*m, Waters Corporation, Milford, MA) by a Waters 2695 system coupled with a Waters 2998 PDA detector. The mobile phase consisted of 0.5% formic acid water solution and acetonitrile. The flow rate was maintained at 0.8 mL·min^−1^, and the injection volume was 20 *µ*L.

### 2.4. Cell Lines and Viruses

Madin–Darby canine kidney (MDCK) epithelial cells were grown in Dulbecco's modified Eagle's medium (DMEM; Cellgro, Manassas, VA, USA) supplemented with 10% fetal bovine serum (FBS; Gibco, Carlsbad, CA, USA), 1,000 units/mL penicillin and 100 *μ*g/mL of streptomycin (Invitrogen, Carlsbad, CA, USA). The replication-competent reporter influenza A virus carrying the *Gaussia* luciferase gene (PR8-PB2-Gluc) and wildtype influenza A/Puerto Rico/8/34 (H1N1, PR8) were propagated as previously described [11, 12, 15]. Infections were performed in Opti-MEM containing 1.5 *µ*g/mL N-tosyl-L-phenylalanine chloromethyl ketone (TPCK)-trypsin (Sigma-Aldrich, St. Louis, MO, USA).

### 2.5. Antiviral Screening

A phenotypic screening for anti-influenza actives were carried out as previously described [11, 16]. In brief, MDCK cells growing in white, flat-bottom, 96-well culture plates (PerkinElmer, Waltham, MA) were infected with PR8-PB2-Gluc virus at 0.01 multiplicities of infection (MOI) in the presence of test samples of 20 *μ*g/mL. After 36-hr incubation, Gluc assay was performed using Pierce Gaussia luciferase glow assay kit (Thermo scientific, Rockford, IL, USA) according to the manufacturer's instructions. Mock infected cells were used as blank control. DMSO and baloxavir acid (BXA) were set as negative and positive control, respectively [17].

### 2.6. Dose-Response Analysis

For dose-response analysis, PR8-PB2-Gluc-infected cells were treated with serially diluted samples, with final concentrations ranging from 40 *μ*g/mL to 0.055 *μ*g/mL. After 36-hrs incubation, the Gaussia luciferase activities were determined.

### 2.7. Cytotoxicity Assay

The cytotoxicity assay was performed as described previously [18]. MDCK cells in 96-well assay plates were treated with serial diluted extracts (two-fold diluted from 200 *μ*g/mL to 1.56 *μ*g/mL for 8 dilution series) and incubated at 37°C for 36 hrs. Cell viability was assessed by CCK-8 (MedChemExpress, Monmouth Junction, NJ, USA) according to the manufacturer's instructions.

### 2.8. Titer Reduction Assay

Titer reduction assay was performed as previously described [11]. In brief, MDCK cells grown in 24-well plates were inoculated with the influenza PR8 virus at an MOI of 0.01. After 2-hour incubation at 37°C, inoculations were replaced by fresh Opti-MEM (1.5 *µ*g/mL of TPCK-trypsin) containing test extracts at 20 *µ*g/mL concentration. At 36-hour after infection (p.i.), the supernatants were removed for titration.

### 2.9. Statistical Analysis

In order to quantify the robustness of the screen, Z′ factor was calculated from the normalized signals from positive and negative control wells on each plate with the following equation: Z′ = 1–3 × (SD of positive control + SD of negative control)/(mean of negative control - mean of positive control). SD represents the standard deviation. Z′ value between 0.5 and 1.0 is considered robust enough for an HTS assay [19].

The percent inhibition of the tested samples was calculated with the following equation: percent inhibition = (signal of negative control–signal of tested compound)/(signal of negative control–signal of positive control) × 100%.

## 3. Results

**Figure 1:**
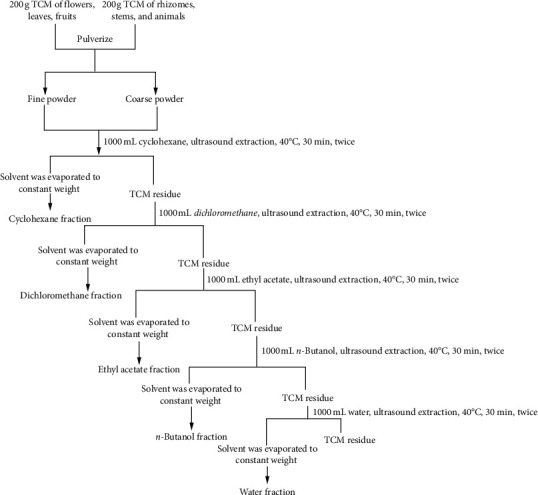
The standard flowchart to extract a traditional Chinese medicinal plant. 100 dried plant materials were extracted, respectively, to obtain various solvent fractions following the method described as mentioned earlier. For each plant medicine, powdered air-dried material (200 g) was extracted with 1000 mL cyclohexane, and the leftover marc was then extracted successively with dichloromethane, ethyl acetate, *n*-butanol, and distilled water by turning the maceration under reflux to obtain the respective fractions. 500 prefractioned extracts were obtained after evaporated in a rotary vacuum evaporator at 40°C to a constant weight of dry extract.

TCMs have proven to possess valuable advantages in clinical practices, including the treatment of influenza virus which caused respiratory disease [20, 21]. Novel anti-influenza actives from TCM samples are therefore anticipated. To this end, 100 medicinal plants which have been recorded as antiviral formula compositions were fractionated with cyclohexane, dichloromethane, ethyl acetate, *n*-butanol, and water sequentially, generating a library consisting of 500 prefractionated TCM extracts (Figure 1, Table S1). As a quality control, three fractions were randomly chosen for re-extraction and HPLC analysis. As Figure S1 shows, the chromatogram of the TCM fraction replicates displayed identical compositions, suggesting that our library is of high quality and reliable for bioactive screen.

**Figure 2:**
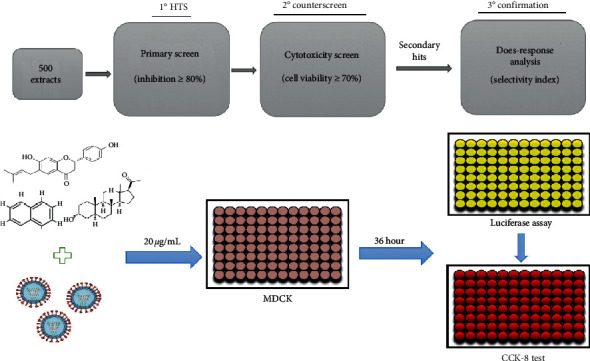
The screening protocol flowcharts. 500 extracts at the concentration of 20 *µ*g/mL were applied to MDCK cells and treated with recombined H1N1 reporter virus for primary screen. Hit compound lists were generated from the primary screen and cytotoxicity screen by applying 80% inhibition and 70% cell viability cutoffs, respectively.

**Figure 3:**
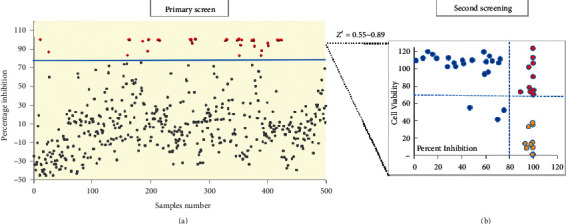
High-throughput phenotypic screening for anti-influenza actives of TCM fractions. (a) Primary screen: percentage inhibition of each fraction was plotted separately. Fractions exhibiting inhibition rate of >80% were colored in red. (b) Secondary screening: preliminarily identified effective hits are tested for the second round of cytotoxicity and confirmation screen. The compounds that showed strong effect on cell viabilities (<70%) were considered as toxic hits (golden dots). Blue dots and red ones represent the inactives and hit fractions, respectively.

Previously, we generated a recombinant IAV expressing *Gaussia* luciferase, based on which a phenotypic high-throughput screening approach was subsequently established, providing a powerful tool for antiviral discovery [11, 12, 15]. A phenotypic screening was therefore carried out against the prefractionated TCM library for anti-influenza actives. The practical screening procedure is shown in Figure 2. Primarily, the inhibitory potency of each TCM fraction at 20 *μ*g/mL against IAV was determined. The Z′ value of each screening plate (ranges from 0.55 to 0.89) was evaluated as quality control (Figure 3(a)). As a result, 47 samples showing >80% inhibition were cherry-picked as primary hits and subjected to a second round of antiviral determination as well as cytotoxicity assay (Figures 2 and 3(a)). Among the 47 primary hits, 13 were excluded due to cytotoxicity, 24 were confirmed as inactives, and the 10 leftover hit fractions were shortlisted for dose-response analysis (Figure 3(b)).

Since all ten hits showed dose-dependent inhibition to IAV replication (Figure S2), the IC_50_ of each hit fraction was therefore calculated, as well as the CC_50_'s and selectivity index (SI) (Table 1). All IC_50_'s of the hits ranges from 1.08 to 6.45 *μ*g/mL. Moreover, except ethyl acetate fraction of *Glycyrrhizic*, of which the SI is lower (7.52), all SIs of the other nine hits exceed 10, suggesting high potency as antiviral actives.

**Figure 4:**
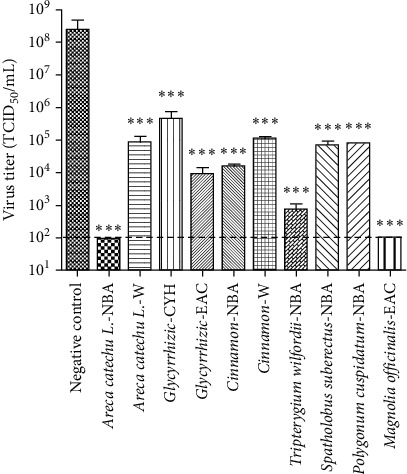
Titer reduction assay against wt-PR8 virus. MDCK cells were infected with influenza PR8 virus in presence of DMSO or indicated samples at 20 *µ*g/mL. At 36 hours after infection, the viral yield was determined. Results are the means ± standard deviation. All data have three multiple duplications. ^∗∗∗^, *P* < 0.0001; ns, no significant difference. The dotted line represents the detection limit.

Considering exogenous luciferase expression was used to indicate IAV replication when using reporter influenza PR8-PB2-Gluc virus, a Gluc inhibitor should probably be identified as false positive. To better address the antiviral activities of hit fractions against IAV, a conventional titer reduction assay were further performed using influenza A/Puerto Rico/8/34 (PR8) virus. As shown in Figure 4, all 10 hit fractions suppressed the viral replication significantly at 20 *μ*g/mL, suggesting that all hit fractions comprise anti-influenza components by targeting influenza viral replication. Notably, NBA fraction of *Areca catechu L*. and EAC fraction of *Magnilia officinalis* almost completely inhibited IAV yielding.

## 4. Discussion

It has been well recognized that, for drug discovery using HTS, diversity within biologically relevant ‘chemical space' is more important than library size, while natural products provide a different, wider, and more drug-like chemical space than synthetic compounds [22, 23]. Moreover, the clinical use of TCMs have proven therapeutic efficacy for various diseases [24–26], coupling the components and specific targets tightly.

In this study, 100 TCM plants were selected as compositions of antiviral prescriptions by literature review [27], and each plant was prepared into 5 simplified extracts, generating a library consisting of 500 TCM fractions (Figure 1, Table S1). Prefractionation can partially remove compounds that are likely to cause artefacts, greatly reducing the complexity of each extract, and as a consequence, increase the hit rate when subjected to an antiviral screening. In addition, since the fractions were prepared by a chromatographic method, subsequent chromatography on a target fraction can be achieved easily, in contrast to the risk that one may fail to find a responsible constituent in active crude extracts [7].

By using a HTS approach based on recombinant reporter influenza PR8-PB2-Gluc virus, the antiviral activity of each fraction against IAV was evaluated, and 10 simplified extracts were identified as anti-influenza actives (Figure 3). All SIs of the hits exceed 10, except with ethyl acetate fraction of Glycyrrhizic, of which the SI is 7.52 (Table 1). It could be proposed that these TCM extracts might contain bioactive components responsible for anti-influenza virus activity at nontoxic concentration, providing a promising source of natural influenza inhibitors.

Interestingly, it was elucidated previously that the antiviral effect of *Polygonum cuspidatum* is associated with active compounds such as resveratrol and emodin, which inhibit the replication of influenza H1N1 virus directly by inducing IFN-*β* [28]. However, *Magnolia officinalis* contains polyphenolic compounds that play a protective role in influenza virus-infected mice [29]. In addition, *Glycyrrhiza*, *Cinnamon*, *Areca catechu L*, and *Spatholobus suberectus* have a wide range of pharmacological activities in clinic, including influenza virus infections [30–33]. These data on the other hand suggested the accuracy and robustness of our antiviral screening results, and the hit fractions deserve further analysis for antiviral discovery.

## 5. Conclusion

In summary, we prepared a library containing 500 prefractionated TCM extracts from 100 herbs with potent antiviral activity. As a pilot, a phenotypic screening was carried out against the library using a recombinant influenza A virus expressing *Gaussia* luciferase, and 10 fractions possessing anti-influenza potencies were identified, including fractions of *Areca catechu L*., *Glycyrrhizin*, *Cinnamon*, *Tripterygium wilfordii*, *Spatholobus suberectus*, *Polygonum cuspidatum*, and *Magnolia officinalis*. It is of great interest to investigate the bioactive components of these extracts and the mechanism of action in future.

## Figures and Tables

**Table 1:** Selectivity indices assay of 10 candidate hits.

TCMs and positive control	Botanical extracts	IC_50_ (*μ*g/mL)^a^	CC_50_ (*μ*g/mL)^b^	SI^c^
*Areca catechu L*.	*n*-Butanol fraction	1.08	50.73	46.97
*Areca catechu L*.	Water fraction	1.27	101.2	79.69
Glycyrrhizin	Cyclohexane fraction	2.12	21.27	10.03
Glycyrrhizin	Ethyl acetate fraction	6.45	48.5	7.52
Cinnamon	*n*-Butanol fraction	1.94	190.9	98.40
Cinnamon	Water fraction	1.95	127.8	65.54
*Tripterygium wilfordii*	*n*-Butanol fraction	1.81	74.43	41.12
*Spatholobus suberectus*	*n*-Butanol fraction	1.99	102.3	51.41
*Polygonum cuspidatum*	*n*-Butanol fraction	1.56	136.2	87.31
*Magnolia officinalis*	Ethyl acetate fraction	3.55	42.94	12.10
BXA	—	0.0015	>0.097	>65

a: IC50, 50% inhibitory concentration; b: CC50, 50% cytotoxicity concentration; c: SI, selectivity index, SI= CC50/IC50. Baloxavir acid as the positive control. The inhibitory and cytotoxicity effects were analyzed using GraphPad Prism 5.

## Data Availability

The data used to support the findings of this study are available from the corresponding author upon reasonable request.

## Supplementary Materials

Supplementary Materials Table S1: the information of the selected 100 plants. Figure S1: comparison of HPLC chromatogram of two *n*-butanol fractions extracted by the same method. Figure S2: the dose-response curves of ten active hits.
