# Supplementary material for: Screening for Anti-Influenza Actives of Prefractionated Traditional Chinese Medicines
Source: Evid Based Complement Alternat Med. 2020 Oct 14;2020:4979850. doi: 10.1155/2020/4979850 (PMC7584957; doi:10.1155/2020/4979850)
Supplement: Supplementary Materials — Table S1: the information of the selected 100 plants. Figure S1: comparison of HPLC chromatogram of two n-butanol fractions extracted by the same method. Figure S2: the dose-response curves of ten active hits. [file 4979850.f1.docx]

**Supplement information**

**Table S1. The selected 100 plants**

| No. | Botanical name | Chinese name | Medicinal parts | Department | Genera |
| --- | --- | --- | --- | --- | --- |
| 1 | Scutellaria barbata D. Don | Banzhilian | Whole plant | Labiatae | Scutellaria L. |
| 2 | Forsythia suspensa (Thunb.) Vahl | Lianqiao | Fruit | Oleaceae | Forsythia Vahl |
| 3 | Sophora flavescens Ait. | Kushen | Root | Leguminosae | Sophora |
| 4 | Sanguisorba officinalis L. | Diyu | Root | Rosaceae | Sanguisorba Linn. |
| 5 | Isatis indigotica Fort. | Banlangen | Root | Brassicaceae Burnett | Isatis |
| 6 | Saposhnikovia divaricata (Trucz.) Schischk. | Fangfeng | Root | Umbelliferae | Saposhnikovia Schischk |
| 7 | Acacia catechu (L.f.) Willd. | Ercha | Branch | Leguminosae | Acacia Mill. |
| 8 | Hedyotis diffusa | Baihuasheshecao | Whole plant | Rubiaceae | Hedyotis Linn. |
| 9 | Pogostemon cablin (Blanco) Benth. | Huoxiang | Whole plant | Labiatae | Agastache Clayt. in Gronov |
| 10 | Isatis indigotica Fort. | Daqingye | Leaves | Brassicaceae Burnett | Isatis |
| 11 | Rhodiola crenulata (Hook. f. et Thoms.) H. Ohba | Hongjingtian | Rhizome and root | Crassulaceae | Rhodiola L. |
| 12 | Sargassum pallidum (Turn.) C. Ag. | Haizao | Frond | Sargassumaceae | Sargassum |
| 13 | Dryopteris crassirhizoma Nakai | Guanzhong | Rhizome | Dryopteridaceae | Dryopteris Adanson |
| 14 | Rosa laevigata Michx. | Jinyingzi | Fruit | Rosaceae | Rose L. |
| 15 | Anemarrhena asphodeloides Bge. | Zhimu | Rhizome | Liliaceae | Anemarrhena Bunge |
| 16 | Paeonia lactiflora PalL | Chishao | Root | Ranunculaceae | Paeonia L. |
| 17 | Xanthium sibiricum Patr. | Cangerzi | Fruit | Asteraceae Bercht. & J. Presl | Xanthium L. |
| 18 | Gentiana scabra Bge. | Longdan | Root | Gentianaceae | Gentiana (Tourn.) L. |
| 19 | Mosla chinensis Maxim. | Xiangru | Whole plant | Labiatae | Elsholtzia |
| 20 | Sonchus oleraceus L. | Beibaijiangcao | Whole plant | Asteraceae Bercht. & J. Presl | Sonchus L. |
| 21 | Mentha haplocalyx Briq. | Bohe | Whole plant and leaves | Labiatae | Mentha Linn. |
| 22 | Schizonepeta tenuifolia Briq. | Jingjie | Stem and leaves， | Labiatae | Nepeta Linn. |
| 23 | Epimedium brevicornu Maxim. | Yinyanghuo | Whole plant | Berberidae | Epimedium Linn. |
| 24 | Lophatherum gracile Brongn. | Danzhuye | Root | Gramineae | Lophatherum |
| 25 | TaraxacummongolicumHand.-Mazz. | Pugongying | Whole plant | Asteraceae Bercht. & J. Presl | Taraxacum F. H. Wigg. |
| 26 | Perilla frutescens (L.) Britt. | Zisuye | Leaves | Labiatae | Perilla Linn. |
| 27 | Perilla frutescens (L.) Britt. | Zisugeng | Stem | Labiatae | Perilla Linn. |
| 28 | Coptis chinensis Franch. | Huanglian | Rhizome | Ranunculaceae | Coptis Salisb. |
| 29 | Lycium chinense Mill. | Digupi | Root bark | Solanaceae | Lycium L. |
| 30 | Imperata cylindrica Beauv.var. Major (Nees) C.E.Hubb. | Baimaogen | Rhizome | Gramineae | Imperata Cyr. |
| 31 | Artemisia scoparia Waldst. et Kit. | Yinchen | Whole plant | Asteraceae Bercht. & J. Presl | Artemisia Linn. Sensu stricto, excl. Sect. Seriphidium Bess. |
| 32 | Pulsatilla chinensis (Bge.) Regel | Baitouweng | Root | Pycnonotidae | Pulsatilla Adans |
| 33 | Salvia miltiorrhiza Bge. | Danshen | Root and rhizome | Labiatae | Salvia Linn. |
| 34 | Gynostemma pentaphyllum (Thunb.) Makino | Jiaogulan | Whole plant | Cucurbitaceae | Gynostemma Bl. |
| 35 | Verbena officinalis L. | Mabiancao | Whole plant | Verbenaceae | Verbena Linn |
| 36 | Curcuma aeruginosa Roxb.[C.zedoaria non Rosc.] | Ezhu | Rhizome | Zingiberaceae | Curcuma L |
| 37 | Ephedra sinica Stapf | Mahuang | Herbaceous stalk | Ephedraceae | Ephedra Tourn.ex L. |
| 38 | PLatycladus orientalis (L.) Franco | Cebaiye | Branches and leaves | Cupressaceae | Platycladus Spach |
| 39 | ArecacatechuL. | Binlang | Seed | Palmae | Areca |
| 40 | Stemona sessilifolia (Miq.)Miq． | Baibu | Root tuber | Centaureae | Angiospermae |
| 41 | Phragmites communis Trin. | Lugen | Rhizome | Gramineae | Phragmites Adans. |
| 42 | CryptotympanapustulataFabricius | Chantui | Rhizome | Cicadidae |  |
| 43 | Glycyrrhiza uralensis Fisch. | Gancao | Root and rhizome | Leguminosae | Glycyrrhiza Linn. |
| 44 | Siphonostegia chinensis Benth. | Beiliujinu | Whole plant | Scrophulariaceae | Siphonostegia |
| 45 | Pueraria lobata (Willd.) Ohwi | Gegen | Root | Leguminosae | Pueraria DC. |
| 46 | Baphicacanthus cusia (Nees) Bremek. | Qingdai | Stem and leaves | Acanthaceae;Brassicaceae Burnett;  Polygonaceae | Isatis;  Baphicacanthus Bremek;  Polygonum L. |
| 47 | Artemisia argyi Levl.et Vant. | Aiye | Leaves | Asteraceae Bercht. & J. Presl | artemisia |
| 48 | Pheretima aspergillum (E.Perrier) | Dilong | Dry body | Megascolecidae |  |
| 49 | Platycodon grandiflorus (Jacq.)A.Dc. | Jiegeng | Root | Campanulaceae | Platycodon A. DC. |
| 50 | Pinellia ternata | Banxia | Tuber | Araceae | Pinellia Tenore |
| 51 | Dolichos lablab L. | Baibiandou | Seed | Leguminosae | Lablab |
| 52 | Punica granatum L. | Shiliupi | Peel | Pomegranaceae | Punica Linn. |
| 53 | Scutellaria baicalensis Georgi | Huangqin | Root | Labiatae | Scutellaria Linn. |
| 54 | Rhus chinensis Mill. | Wubeizi | Galls | Lacqueraceae | Rhus (Tourn.) L. emend. Moench |
| 55 | Cinnamomum cassia Presl | Rougui | Bark | Lauraceae | Cinnamomum Trew |
| 56 | Massa Medicated Fermentata | Shenqu |  |  |  |
| 57 | Phellodendron amurense Rupr. | Huangbai | Whole plant | Rutaceae | Clausena Burm. f. |
| 58 | Lindera aggregata (Sims) Kos-term. | Wuyao | Root | Lauraceae | Lindera Thunb. |
| 59 | Pyrrosia sheareri (Bak.) Ching | Shiwei | Leaves | Polypodiaceae | Pyrrosia Mirbel |
| 60 | Lysimachia christinae Hance | Jinqiancao | Whole plant | Primulaceae | Lysimachia |
| 61 | Astragalus membranaceus (Fisch.) Bge. | Huangqi | Root | Leguminosae | Astragalus Linn. |
| 62 | Angelica sinensis (Oliv.) Diels | Danggui | Root | Umbelliferae | Angelica L |
| 63 | Poria cocos (Schw.) Wolf | Fuling | Dry sclerotia of fungi | Porphyridae | Wolfiporia Ryv.&Gilbn |
| 64 | ScolopendrasubspinipesmutilansL.Koch | Wugong | Dry body |  |  |
| 65 | Atractylodes lancea (Thunb.) DC. | Cangzhu | Rhizome | Asteraceae Bercht. & J. Presl | Atractylodes DC. |
| 66 | Paris polyphylla Smith var. chinensis (Franch.) Hara | Chonglou | Rhizome | Liliaceae | Paris L. |
| 67 | Spirodela polyrrhiza (L.) Schleid. | Fuping | Whole plant | Lemnaceae | Lemna L. |
| 68 | Polygonum multiflorum Thunb. | Heshouwu | Root | Polygonaceae | Fallopia Adans. |
| 69 | Carthamus tinctorius L. | Honghua | Flowers | Asteraceae Bercht. & J. Presl | Carthamus |
| 70 | Tripterygium wilfordii Hook. f. | Leigongteng | Woody stem | Celastraceae | Tripterygium wilfordii Hook. f. |
| 71 | Polygonum cuspidatum Sieb.et Zucc. | Huzhang | Root and rhizome | Polygonaceae | Reynoutria Houtt. |
| 72 | Polygonatum kingianum Coll.etHemsl. | Huangjing | Rhizome | Liliaceae | Polygonatum Mill. |
| 73 | Lonicera japonica Thunb. | Jinyinhua | Flowers | Lonicerae | Lonicera Linn |
| 74 | Chrysanthemum morifolium Ramat. | Juhua | Flowers | Asteraceae Bercht. & J. Presl | Dendranthema (DC.) Des Moul. |
| 75 | Brucea javanica (L.) Merr. | Yadanzi | Fruit | Simarubaceae | Brucea J. F. Mill. |
| 76 | Geranium wilfordii Maxim. | Laoguancao | Whole plant | Geraniaceae | Geranium L. |
| 77 | Belamcanda chinensis (L.) DC. | Shegan | Rhizome | Iridaceae | Belamcanda Adans |
| 78 | VitextrifoliaL.Var.simplicifoliaCham. | Manjingzi | Fruit | Verbenaceae | Vitex L. |
| 79 | Paeonia suffruticosa Andr. | Mudanpi | Root bark | Ranunculaceae | Paeonia L. |
| 80 | Spatholobus suberectus Dunn | Jixueteng | Rattan | Leguminosae | Spatholobus Hassk. |
| 81 | Lasiosphaera fenzlii Reich | Mabo | Fruiting body | Basidiomycota |  |
| 82 | Fraxinus rhynchophylla Hance | Qinpi | Bark | Oleaceae | Fraxinus L |
| 83 | Artemisia annua L. | Qinghao | Above ground plant | Asteraceae Bercht. & J. Presl | Artemisia Linn. Sensu stricto, excl. Sect. Seriphidium Bess. |
| 84 | Panax notoginseng (Burk.) F.H.Chen | Sanqi | Root and rhizome | Acanthopanaceae | Panax L. |
| 85 | Magnolia officinalis Rehd. et Wils. | Houpo | Stem and root bark | Magnoliaceae | Magnolia L. |
| 86 | Portulaca oleracea L. | Machixian | Whole plant | Portulaceae | Portulaca L. |
| 87 | Cimicifuga heracleifolia Kom. | Shengma | Rhizome | Ranunculaceae | Cimicifuga |
| 88 | Bubablus bubalis Linnaeus | Shuiniujiao | Horn |  |  |
| 89 | Asparagus cochinchinensis (Lour.) Merr. | Tiandong | Root | Liliaceae | Asparagus L. |
| 90 | Arisaema erubescens (Wall.) Schott | Tiannanxing | Stem | Araceae | Arisaema Mart. |
| 91 | Prunus mume (Sieb.) Sieb. et Zucc. | Wumei | Fruit | Rosaceae | Armeniaca Mill. |
| 92 | siegesbeckiaorientalisL. | Xixiancao | Above ground plant | Asteraceae Bercht. & J. Presl | Siegesbeckia |
| 93 | Prunella vulgaris L. | Xiakucao | Fruit | Labiatae | Prunella |
| 94 | Magnolia biondii Pamp. | Xinyi | Fruit | Magnoliaceae | Magnolia L |
| 95 | Scrophularia ningpoensis Hemsl. | Xuanshen | Root | Scrophulariaceae | Scrophularia L. |
| 96 | Aster tataricus L.f. | Ziwan | Root and rhizome | Asteraceae Bercht. & J. Presl | Aster |
| 97 | Gentiana macrophylla Pall. | Qinjiao | Root | Gentianaceae | Gentiana (Tourn.) L. |
| 98 | Polyporus umbellatus (Pers.) Fries | Zhuling | Under ground sclerotia | Porphyridae | Polyporus |
| 99 | Arnebia euchroma (Royle) Johnst. | Zicao | Root | Arnebiaceae | Lithospermum L. |
| 100 | Houttuynia cordata Thunb. | Yuxingcao | Above ground plant | Sanbaicaceae | Houttuynia Thunb. |


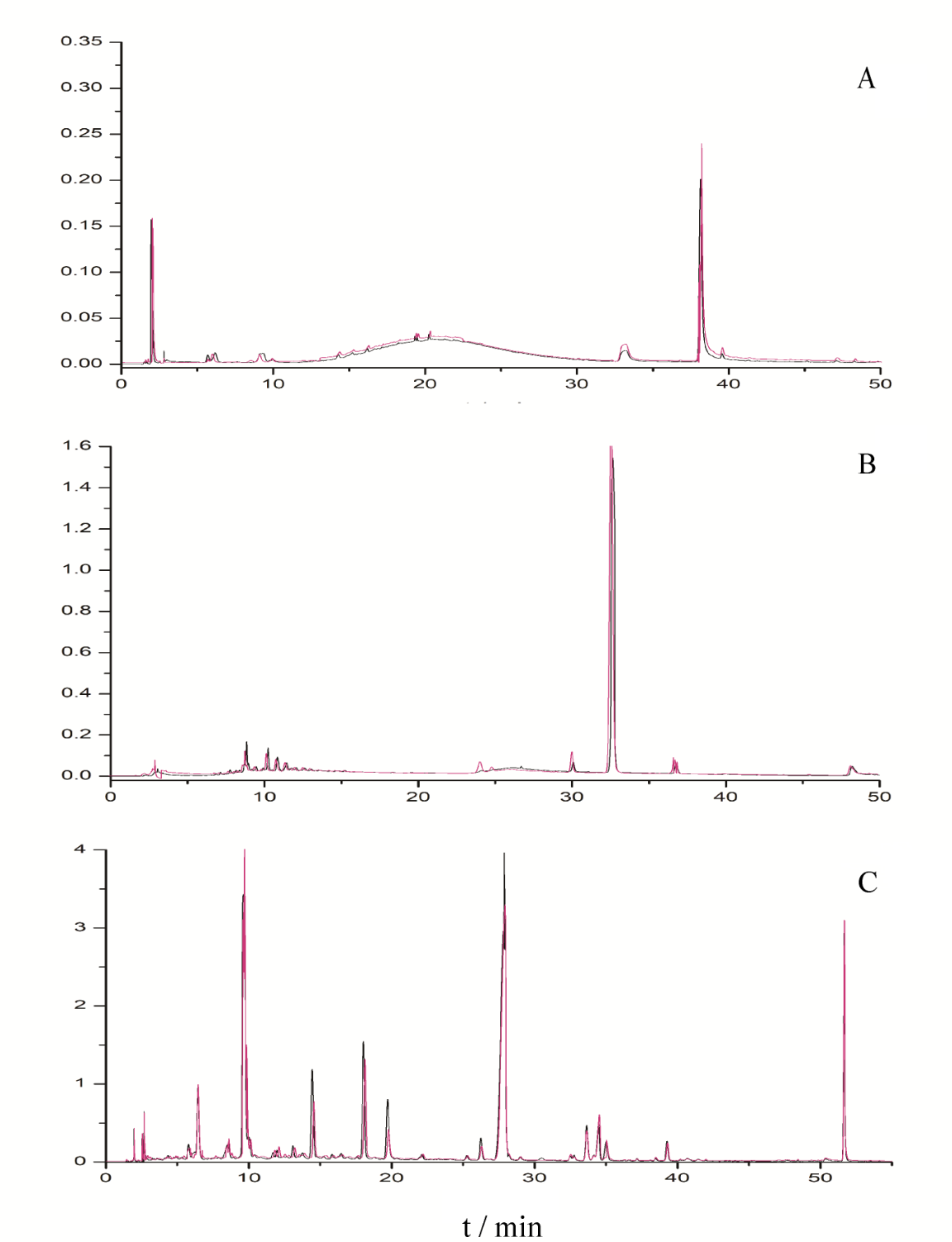


**Figure S1. Comparison of HPLC chromatogram of two n-butanol fractions extracted by the same method.** A) HPLC chromatogram of the n-butanol fractions of *Areca catechu L*. B) HPLC chromatogram of the n-butanol fractions of *cinnamon*. C) HPLC chromatogram of the n-butanol fractions of *Polygonum cuspidatum*. The first extractives of three pre-distillates were represented by black curves, and the red lines indicate the second extracted collections.


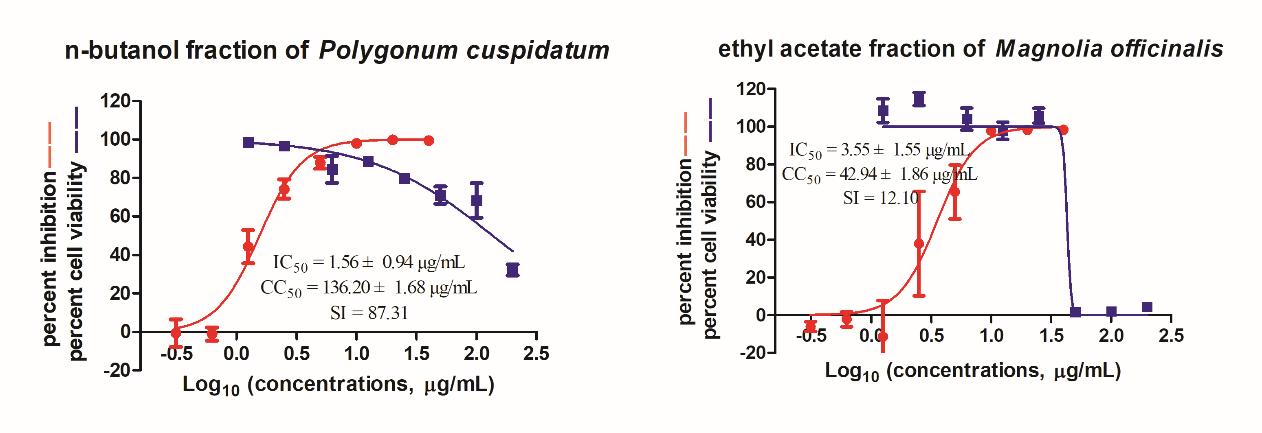

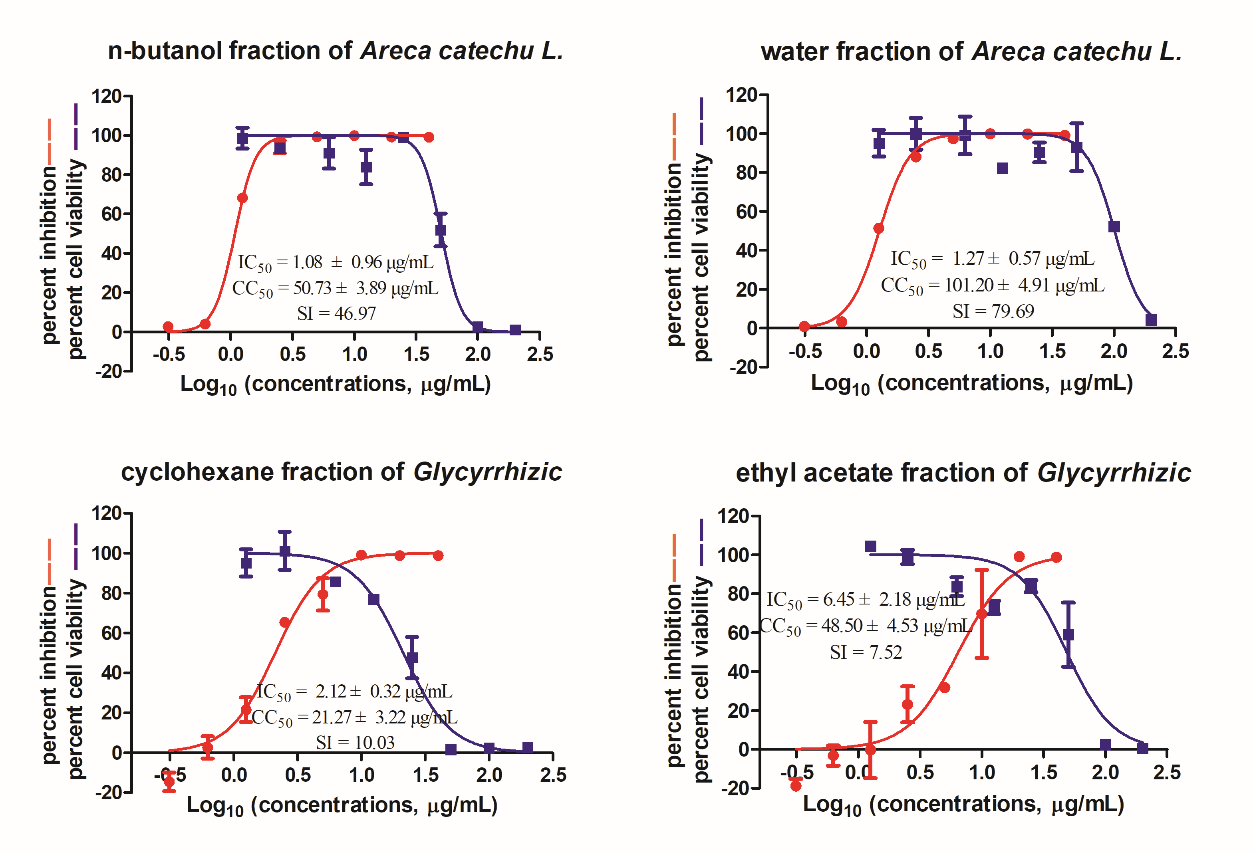

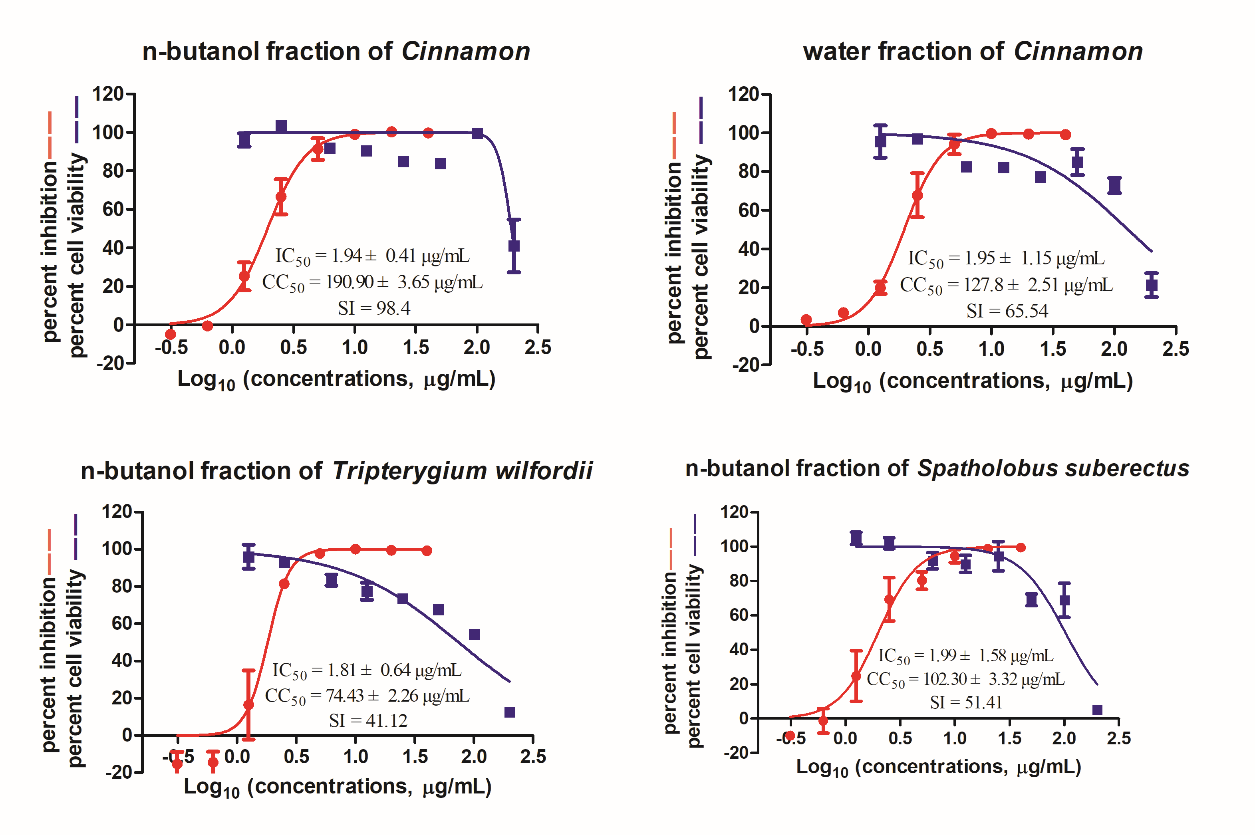


**Figure S2. The dose-response curves of ten active hits.** Each fraction was diluted in an eight-point concentration and exhibited a dose-dependent manner of inhibition. Data are means ± SD from three independent experiments.
